# Comparison of endoscopic features of early-stage squamous cell lung cancer and histological findings

**DOI:** 10.1038/sj.bjc.6690540

**Published:** 1999-07

**Authors:** C Konaka, T Hirano, H Kato, K Furuse, M Takada, Y Saito, Y Monden, E Matsui, Y Watanabe

**Affiliations:** Department of Surgery, Tokyo Medical University, 6-7-1 Nishishinjuku Shinjuku-ku, Tokyo, 160-0023, Japan; Department of Internal Medicine, National Kinki Central Hospital for Chest Disease, Osaka, Japan; Department of Internal Medicine, Osaka Prefectur Habikino Hospital, Osaka, Japan; Institute of Development, Aging and Cancer, Tohoku University, Sendai, Japan; Department of Surgery, The University of Tokushima, School of Medicine, Tokushima, Japan; Department of Radiology, Gifu University, School of Medicine, Gifu, Japan; Department of Surgery, Kanazawa University, School of Medicine, Kanazawa, Japan

**Keywords:** early-stage central-type of squamous cell carcinoma, endoscopic features, histopathological findings, bronchus

## Abstract

Seventy cases with early-stage central-type squamous cell carcinoma were treated surgically between 1984 and 1993 in seven participating institutes. We classified endoscopic features of early-stage central-type squamous cell carcinoma into three types (hypertrophic type, nodular type and polypoid type). After surgery we investigated the relationship between endoscopic features and both the area of superficial extent and depth of carcinoma invasion based on histopathological investigations of the surgical specimens. In 66.7% of the hypertrophic type lesions cancer cells did not invade into the cartilaginous layer, and only 4.8% of this type showed tumour invasion beyond the bronchial cartilage. On the other hand, a few nodular and polypoid type cases showed in-situ carcinoma or carcinoma with invasion from the subepithelial layer to the muscle layer, and in approximately 20% the these types we observed carcinoma invasion beyond the cartilaginous layer, which was not suitable for photodynamic therapy. Also, concerning the greatest dimension 24 out of 35 lesions (68.6%) less than 10 mm in the greatest dimension were evaluated as either in-situ carcinoma or micro-invasive tumour within the muscle layer. The endoscopic features can provide a basis for the determination of therapeutic strategy in early-stage central-type lung cancer. © 1999 Cancer Research Campaign


*British Journal of Cancer* (1999) **80**(9), 1435–1439
© 1999 Cancer Research Campaign
Article no. bjoc.1998.0540

### Comparison of endoscopic features of early-stage squamous cell lung cancer and histological findings

**C Konaka[1], T Hirano[1], H Kato[1], K Furuse[2], M Takada[3], Y Saito[4], Y Monden[5], E Matsui[6] and Y Watanabe[7]**

[1]Department of Surgery, Tokyo Medical University, 6-7-1 Nishishinjuku Shinjuku-ku, Tokyo 160-0023, Japan; [2]Department of Internal Medicine, National Kinki Central Hospital for Chest Disease, Osaka, Japan; [3]Department of Internal Medicine, Osaka Prefectur Habikino Hospital, Osaka, Japan; [4]Institute of Development, Aging and Cancer, Tohoku University, Sendai, Japan; [5]Department of Surgery, The University of Tokushima, School of Medicine, Tokushima, Japan; [6]Department of Radiology, Gifu University, School of Medicine, Gifu, Japan; [7]Department of Surgery, Kanazawa University, School of Medicine, Kanazawa, Japan

**Summary** Seventy cases with early-stage central-type squamous cell carcinoma were treated surgically between 1984 and 1993 in seven participating institutes. We classified endoscopic features of early-stage central-type squamous cell carcinoma into three types (hypertrophic type, nodular type and polypoid type). After surgery we investigated the relationship between endoscopic features and both the area of superficial extent and depth of carcinoma invasion based on histopathological investigations of the surgical specimens. In 66.7% of the hypertrophic type lesions cancer cells did not invade into the cartilaginous layer, and only 4.8% of this type showed tumour invasion beyond the bronchial cartilage. On the other hand, a few nodular and polypoid type cases showed in-situ carcinoma or carcinoma with invasion from the subepithelial layer to the muscle layer, and in approximately 20% the these types we observed carcinoma invasion beyond the cartilaginous layer, which was not suitable for photodynamic therapy. Also, concerning the greatest dimension 24 out of 35 lesions (68.6%) less than 10 mm in the greatest dimension were evaluated as either in-situ carcinoma or micro-invasive tumour within the muscle layer. The endoscopic features can provide a basis for the determination of therapeutic strategy in early-stage central-type lung cancer.

**Keywords:** early-stage central-type of squamous cell carcinoma; endoscopic features; histopathological findings; bronchus

The introduction of sputum cytology screening in mass surveys has increased the detection of roentgenologically occult lung cancer (Matsuda et al, 1990). When severely atypical cells are detected in the sputum of subjects with normal roentgenographical findings, bronchoscopy should be performed, because most roentgenologically occult cancers, which are squamous cell carcinoma (SCC) histopathologically, are located in relatively large bronchi (Saito et al, 1992). Most roentgenologically occult cancers invade within the bronchial wall but are not accompanied by metastasis. In general, we classify such lesions as early-stage squamous cell carcinoma. However, it is difficult to evaluate the depth of intrabronchial invasion only from endoscopic features. Furthermore, the area of extent is small, and multicentric occurrence is frequently observed either synchronously, metachronously or both (Broghamer et al, 1985; Rosengart et al, 1991). Therefore, detailed observation based on extensive clinical experience in bronchoscopy is needed. However, the endoscopic criteria of early-stage central-type SCC remain to be clarified, especially concerning the relationship between endoscopic features and depth of intrabronchial invasion.

Some reports indicate that endoscopic laser photodynamic therapy (PDT) (Okunaka et al, 1991) and brachytherapy (Saito et al, 1996) can yield curative results in early-stage central-type lung cancer. From the standpoint of both multicentric occurrence and high frequency in heavy smokers with poor pulmonary function, endoscopic laser therapy may be superior to surgical resection. However, in the case of endoscopic therapy there is a high possibility of residual lesions or local recurrence unless endoscopically accurate evaluation can be made of both the area of extent and depth of tumour invasion. Therefore, the establishment of endoscopic criteria of early-stage SCC is very important for the determination of appropriate therapeutic strategy.

In this retrospective study in which seven institutes participated, the authors attempted to clarify the relationship between endoscopic features and both the extent and the depth of intrabronchial invasion based on histopathological evaluation of surgically resected specimens.

*Received 27 July 1998*
*Revised 10 December 1998*
*Accepted 22 December 1998*

*Correspondence to:* C Konaka

### PATIENTS AND METHODS

Seventy patients with roentgenologically occult cancer, who were histopathologically diagnosed as early-stage central-type of squamous cell lung carcinoma, were resected between 1984 and 1993 at the seven institutes. We defined central-type lung cancer as tumour localization in the segmental or more proximal bronchi. Furthermore, we defined early-stage central-type squamous cell lung carcinoma as a lesion limited to within the bronchial wall without any metastasis. In these 70 cases we evaluated the relationship between endoscopic features and both the superficial extent and the depth of cancer invasion, using surgically resected specimens.

### Patient characteristics

Early-stage central-type SCC were resected in 70 patients (67 men and three women). The age of the patients ranged from 52 to 78 (average age 67.8). Five lesions were located in main bronchi or the truncus intermedius, and four lesions were located in lobar

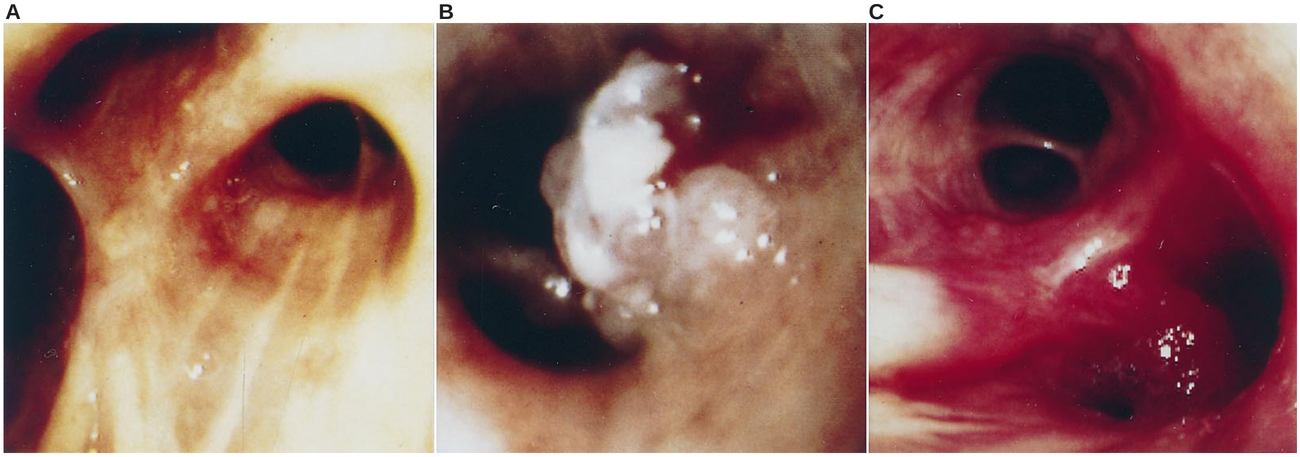

**Figure 1** (**A**) Endoscopic features of a representative hypertrophic-type lesion. The lesion is located at the bifurcation of right $B^1$ and $B^2$. Thickened epithelium and granular changes are observed on the surface of the spur. (**B**) Representative endoscopic features of a nodular-type lesion. The lesion is located at the orifice of the left basal bronchus. Approximately 4 mm elevation of bronchial mucosa with a large basement is observed. (**C**) Representative endoscopic features of a polypoid-type lesion. The lesion is located at the bifurcation of right $B^6$a and $B^6$b. In this pedunculated tumour respiratory movement is observed on respiration

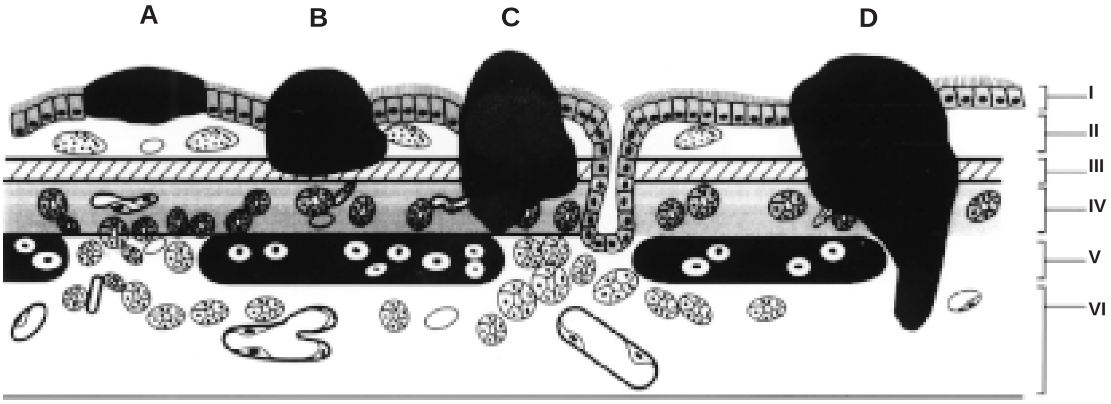

**Figure 2** This schematic illustration shows the intrabronchial structure. I: epithelial layer, II: subepithelial layer, III: muscle layer, IV: extramuscle layer, V: cartilaginous layer, VI: extracartilaginous layer. (**A**) In-situ carcinoma; (**B**) micro-invasive tumour within the muscle layer; (**C**) micro-invasive tumour within the cartilaginous layer; (**D**) invasive tumour beyond the cartilaginous layer bronchi. Though the residual 61 lesions originated in segmental bronchi, it was possible to recognize the peripheral margin in all lesions bronchoscopically. There was no case of synchronous multicentric occurrence.

### Endoscopic features of early-stage central-type squamous cell lung carcinoma

We classified the endoscopic findings of early-stage central-type squamous cell lung carcinoma into three types (hypertrophic type, nodular type and polypoid type).

*Hypertrophic type*

The bronchial epithelium is superficially elevated. This type of lesion is frequently seen at bifurcations. Representative features of this type are shown in Figure 1A. It can be difficult to distinguish some lesions of this type from normal mucosa endoscopically, but thickened epithelium and granular changes can usually be observed on the surface of the bronchial mucosa.

*Nodular type*

This type of lesion has elevation of bronchial mucosa with a large base. The height of the elevated bronchial mucosa is 2 mm or more. Representative features are shown in Figure 1B.

*Polypoid type*

This lesion shows a pedunculated tumour. Sometimes respiratory movement is observed on respiration. Representative features are shown in Figure 1C.

### Histopathological evaluation using resected specimens

The bronchial wall containing the cancerous lesion was serially cut at a thickness of 3 mm and the superficial extent and depth of the tumour invasion were evaluated using sectioned specimens stained by haematoxylin and eosin. The area of extent was measured in terms of both the longitudinal length along the bronchial axis and the greatest dimension in one lesion based on observation of serially cut specimens, microscopically.

© 1999 Cancer Research Campaign

Simultaneously, the degree of intrabronchial invasion was evaluated microscopically, using sectioned specimens. Based on the deepest area of invasion, we defined the tumour as in-situ carcinoma, carcinoma with invasion from the subepithelial layer to the muscle layer (micro-invasive tumour within the muscle layer), carcinoma with invasion from the extramuscle layer to the cartilaginous layer (micro-invasive tumour within the cartilaginous layer) and carcinoma with invasion beyond the cartilaginous layer without extrabronchial development (invasive tumour beyond the cartilaginous layer) (Figure 2).

### Statistical eveluation

Statistical evaluation of data was performed using Cochran–Mantel–Haenszel Statistics (Mantel et al, 1959). A $P$-value of less than 0.01 was taken to indicate a significant difference.

### RESULTS

On preoperative endoscopy, 42 lesions (60.0%) were classified as hypertrophic type, 17 lesions (24.3%) as nodular type while the frequency of polypoid type was relatively low (11 lesions, 15.7%). Detailed clinicopathological characteristics are listed in Table 1.

The relationship between endoscopic features and the greatest dimension of lesions is shown in Table 2. The greatest dimension of the 70 lesions varied from 2 to 50 mm (average, 11.8 mm), with 77.8% of the lesions being 4 mm or less in greatest dimension and 76.1% of lesions 5–9 mm in greatest dimension belonging to the hypertrophic type bronchoscopically. On the other hand, in lesions 10-mm or more in greatest dimension, the frequency of nodular and polypoid types increased. There was a statistically significant relationship between endoscopic features and the greatest dimension of lesions ($P < 0.001$).

The relationship between endoscopic features and the depth of intrabronchial invasion is shown in Table 3. In the hypertrophic type, 50.0% and 16.7% revealed in-situ carcinoma and micro-invasive tumour within the muscle layer respectively. Only one nodular type lesion was in-situ carcinoma. Also, only one polypoid type lesion revealed micro-invasive tumour within the muscle layer. Most cases of nodular and polypoid types showed intra-cartilaginous invasion. Furthermore, three lesions (17.6%) of the nodular type and three lesions (27.3%) of the polypoid type revealed invasive tumour beyond the cartilaginous layer. A statistically significant relationship between endoscopic features and the depth of intrabronchial invasion was recognized ($P < 0.001$).

Table 4 shows the relationship between the greatest dimension in lesions and the depth of intrabronchial invasion. In lesions 4-mm or less in greatest dimension, 77.8% showed either in-situ carcinoma or micro-invasive tumour within the muscle layer. On the other hand, 82.9% of lesions 10 mm and more in greatest diameter invaded either the cartilaginous layer or the extracartilaginous layer. There was a statistically significant relationship between the greatest dimension and the depth of intrabronchial invasion ($P < 0.001$).

**Table 1** Clinical data of patients with early-stage central-type squamous cell lung cancer

| **Characteristics** | ***n*** |
|---|---|
| No. of patients | 70 |
| Sex (male:female) | 67:3 |
| Average age (year) | 52–78 (Average 67.8) |
| Tumour localization | |
| Trachea | 0 |
| Main bronchus | 3 |
| Truncus intermedius | 2 |
| Lobar bronchi | 4 |
| Segmental bronchi | 61 |
| The greatest dimension of the tumour (mm) | 2–50 (Average 11.8) |

**Table 2** Relationship between endoscopic features and the greatest dimension of the lesion

| | –4 mm | 5–9 mm | 10–19 mm | 20 mm+ | Total |
|---|---|---|---|---|---|
| Hypertrophic-type | 14 (33.3%) | 13 (31.0%) | 5 (11.9%) | 10 (23.8%) | 42 |
| Nodular-type | 1 (5.9%) | 2 (11.8%) | 7 (41.2%) | 7 (41.2%) | 17 |
| Polypoid-type | 3 (27.3%) | 2 (18.2%) | 4 (36.4%) | 2 (18.2%) | 11 |
| Total cases | 18 (25.7%) | 17 (24.3%) | 16 (22.9%) | 19 (27.1%) | 70 |

$P < 0.001$.

### DISCUSSION

Most central-type lung cancer cases with roentgenographically abnormal findings show either extrabronchial invasion, regional lymph node involvement, or both. If the lesion is allowed to progress to the invasive stage, prognosis is poor. Unfortunately, most patients with primary lung cancer have advanced disease at the initial diagnosis. The 5-year survival rate of patients with primary lung cancer is estimated to be less than 15% (Ries et al, 1983). At present, the outcome of treatment of primary lung cancer can only be improved by efforts to discover early-stage lung cancer. In the peripheral type, it is easy to detect even small tumour shadows roentgenographically. However, in central-type cases early-stage lesions were always roentgenographically occult.

**Table 3** Relationship between endoscopic features and depth of interbronchial invasion

| | In-situ carcinoma | Micro-invasive tumour within the muscle layer | Micro-invasive tumour within the cartilaginous layer | Invasive tumour within the bronchial wall | Total cases |
|---|---|---|---|---|---|
| Hypertrophic-type | 21 (50.0%) | 7 (16.7%) | 12 (28.6%) | 2 (4.8%) | 42 |
| Nodular-type | 1 (5.9%) | 0 (0%) | 13 (76.5%) | 3 (17.6%) | 17 |
| Polypoid-type | 0 (0%) | 1 (9.1%) | 7 (63.6%) | 3 (27.3%) | 11 |
| Total cases | 22 (31.4%) | 8 (11.4%) | 32 (45.7%) | 8 (11.4%) | 70 |

$P < 0.001$.

**Table 4** Relationship between the greatest dimension of the lesion and depth of interbronchial invasion

| | In-situ carcinoma | Micro-invasive tumour within the muscle layer | Micro-invasive tumour within the cartilaginous layer | Invasive-tumour within the bronchial wall | Total cases |
|---|---|---|---|---|---|
| –4 mm | 11 (61.1%) | 3 (16.7%) | 3 (16.7%) | 1 (5.6%) | 18 |
| 5–9 mm | 9 (52.9%) | 1 (5.9%) | 5 (29.4%) | 2 (11.8%) | 17 |
| 10–19 mm | 0 (0%) | 1 (6.3%) | 13 (81.3%) | 2 (12.5%) | 16 |
| 20 mm + | 2 (10.5%) | 3 (15.8%) | 11 (57.9%) | 3 (15.8%) | 19 |
| Total cases | 22 (31.4%) | 8 (11.4%) | 32 (45.7%) | 8 (11.4%) | 70 |

$P < 0.001$.

Therefore, both sputum cytology and bronchoscopy are needed for early detection in central-type lung cancer, and it is impossible to determine the localization of the lesion without endoscopically detailed observation.

Although most nodular type or polypoid type lesions are grossly visible endoscopically, it is often difficult to detect hypertrophic-type lesions, which constitute 60% of central-type early-stage lung cancer. Slight changes in bronchial mucosa must not be overlooked. Some parts of this type of lesion are recognized at bronchial bifurcations as either thickening or enlargement of the bifurcation. This type of lesion located at sites other than bronchial bifurcations can be more difficult to detect, and the only findings may be reduction of lustre or granular changes on the surface of the bronchial mucosa.

Since 1980 we have treated lesions that were endoscopically suggestive of early-stage squamous cell carcinoma by PDT, if the peripheral margin was recognizible. In this treatment, irradiation of 630-nm light generated by an argon dye or excimer laser is performed after intravenous injection of a photosensitizer. PDT is conservative therapy to preserve lung tissue and we succeeded in achieving cure in many cases with early-stage central type SCC (Kato et al, 1986). However, there is little likelihood that the 630-nm laser beam would penetrate beyond the bronchial cartilage. Therefore, it is essential to endoscopically evaluate the area of extent and the depth of intrabronchial invasion accurately before PDT.

From the standpoint of therapeutic strategy, it is very important to evaluate the relationship between the endoscopic features and tumour invasion. However, few reports referred to the endoscopic criteria of early-stage central-type lung cancer (Akaogi et al, 1994). In this context, we attempted to classify the endoscopic features of early-stage central-type squamous cell lung carcinoma, and also to clarify the relationships between endoscopic features and histopathologically recognized tumour invasion in this study.

In the hypertrophic type, 66.7% showed in-situ carcinoma or micro-invasion within the muscle layer. These cases would be suitable for PDT. However, two out of 42 hypertrophic-type lesions (4.8%) revealed invasion beyond the cartilaginous layer. In these two lesions remarkable granular changes were observed on the surface of the bronchial mucosa, and their greatest dimensions were both more than 20 mm. All except one nodular type lesions had either micro-invasion within the cartilaginous layer or invasion beyond the cartilaginous layer. One nodular type lesion 5 mm in greatest dimension was evaluated as in-situ carcinoma. Also, only one polypoid-type lesion which was 3-mm in the greatest dimension was diagnosed as micro-invasive tumour within the muscle layer. The other polypoid lesions invaded the cartilaginous or deeper layers.

In the analysis based on the greatest dimension, 24 out of 35 lesions (68.6%) less than 10 mm in greatest dimension were either in-situ carcinomas or micro-invasive tumours within the muscle layer. On the other hand, in 35 lesions with 10 mm or more in greatest dimension, only six lesions (17.1%) did not invade the cartilaginous layer.

The present data suggest that there is a strong possibility that hypertrophic type lesions, less than 10 mm in greatest dimension are either in-situ carcinoma or micro-invasive tumour within the muscle layer. In nodular-type and polypoid-type and lesions 10 mm or more in greatest dimension there is little chance that invasion is only within the muscle layer.

It appears that PDT is indicated in hypertrophic type lesions less than 10 mm in greatest dimension. We have attempted to treat tumours with micro-invasion within the cartilaginous layer by PDT. However, before treatment we cannot point to any definitive endoscopic features that allow us to distinguish between tumours with only micro-invasion within the cartilaginous layer and tumours with invasion beyond the cartilaginous layer. In this study we measured the greatest dimension in resected specimens microscopically and recognized that there is some discrepancy between endoscopic evaluation and the actual area of tumour extent.

Recently, fluorescence bronchoscopy techniques are now used clinically to localize small lesions and to accurately determine the area of extent (Lam et al, 1994). Also, endoscopic ultrasound can be useful to evaluate the depth of tumour invasion into the bronchial wall and the adjacent structures (Schuder et al, 1991; Hurther et al, 1992). In the near future these examinations will become essential for endoscopic diagnosis of central-type lung cancer. However, at present these systems have not yet become standard tools for the detection of roentgenologically occult cancer, because we cannot accurately evaluate the images of fluorescence bronchoscopy and endoscopic ultrasound without the further accumulation of clinical experience. Furthermore, even though fluorescence bronchoscopy is available, it can be impossible to distinguish the hypertrophic type of early-stage central-type lung cancer from squamous cell metaplasia, dysplasia or basal cell hyperplasia. In this context, re-evaluation of the endoscopic features of early-stage central-type of SCC is very important.

In all 70 cases we evaluated in this study 5-year survival data were already investigated. In six cases – two cases (4.77%) of hypertrophic type, one case (5.88%) of nodular type and three cases (27.3%) of polypoid type – we could confirm death caused by the cancerous lesions. The outcome of both hypertrophic type and nodular type was markedly superior to stage I invasive SCC of

© 1999 Cancer Research Campaign the bronchus. However, we could not recognize a significant difference in 5-year survival between the polypoid-type and stage I invasive SCC of the bronchus. Five cases out of six polypoid-type cases showed either micro-invasive tumour within the cartilaginous layer or invasive tumour beyond the cartilaginous layer. Therefore, we think that it is important to detect hypertrophic type and nodular type by careful endoscopic observation.

In conclusion, we classified endoscopic findings into three types based on endoscopic features. The possibility of invasion beyond the cartilaginous layer was higher in nodular and polypoid types than in the hypertrophic type. The combined evaluation of endoscopic features and the greatest dimension of the lesion provide some basis for evaluation of the degree of tumour development.

### ACKNOWLEDGEMENTS

The authors thank Professor J Patrick Barron of the International Medical Communication Center of Tokyo Medical University for his review of the manuscript.

### REFERENCES

Akaogi E, Ogawa I, Mitsui K, Onizuka M, Ishikawa S, Yamamoto T, Inage Y and Ogata T (1994) Endoscopic criteria of early squamous cell carcinoma of the bronchus. *Cancer* **74**: 3113–3117

Broghamer WL, Richards ME, Biscopink RJ and Faurest SH (1985) Pulmonary cytologic examination in the identification of the second primary carcinoma of the lung. *Cancer* **56**: 2664–2668

Hurther TH and Hanrath P (1992) Endobronchial sonography: feasibility and preliminary results. *Thorax* **47**: 565–567

Kato H, Konaka C, Kawate N, Shinohara H, Kinoshita K, Noguchi M, Ootomo S and Hayata Y (1986) Five-year disease-free survival of a lung cancer patient treated only by photodynamic therapy. *Chest* **90**: 768–770

Lam S, MacAulay C, Hung J, LeRiche J and Palcic B (1993) Detection of dysplasia and carcinoma in-situ using a lung imaging fluorescence endoscope (LIFE) device. *J Thorac Cardiovasc Surg* **105**: 1035–1040

Mantel N and Haenszel W (1959) Statistical aspects of the analysis of data from retrospective studies of disease. *J Natl Cancer Inst* **22**: 719–748

Matsuda M, Horai T, Doi O, Kodama K and Tateishi R (1990) Diagnosis of squamous-cell carcinoma of the lung by sputum cytology: with special reference to correlation of diagnostic accuracy with size and proximal extent of resected tumor. *Diagn Cytopathol* **6**: 248–251

Okunaka T, Kato H, Konaka C, Kawate N, Bonaminio A, Yamamoto H, Ikeda N, Tolentino M, Eckhauser ML and Hayata Y (1991) Photodynamic therapy for multiple primary bronchogenic carcinoma. *Cancer* **68**: 253–258

Ries LG, Pollack ES and Young JL (1983) Cancer patient survival: surveillance, epidemiology and end results program. *J Natl Cancer Inst* **70**: 693–707

Rosengart TK, Martini N, Ghosn P and Burt M (1991) Multiple primary lung carcinoma: prognosis and treatment. *Ann Thorac Surg* **52**: 773–779

Saito M, Yokoyama A, Kurita Y, Uematsu T, Miyano H and Fujimori K (1996) Treatment of roentgenographically occult endobronchial carcinoma with external beam radiotherapy and intraluminal low dose rate brachytherapy. *Int J Radiat Oncol Biol Phys* **34**: 1029–1035

Saito Y, Nagamoto M, Ota S, Sagawa M, Kamma K, Takahashi S, Usuda K, Endo C, Imai T and Fujimura S (1992) Results of surgical treatment for roentgenographically occult bronchogenic squamous cell carcinoma. *J Thorac Cardiovasc Surg* **104**: 401–407

Schuder G, Isringhaus H, Kubale B, Seitz G and Sybrecht GW (1991) Endoscopic ultrasonography of the mediastinum in the diagnosis of bronchial carcinoma. *Thorac Cardiovasc Surg* **39**: 299–303

*© 1999 Cancer Research Campaign*
